# Differential Risk Factors for Early Intravesical Recurrence After Radical Nephroureterectomy for Upper Urinary Tract Carcinoma According to the History of Non‐Muscle Invasive Bladder Cancer

**DOI:** 10.1111/iju.70009

**Published:** 2025-02-13

**Authors:** Tetsuya Shindo, Yohei Ueki, Ippei Muranaka, Genki Kobayashi, Shintaro Miyamoto, Yasuharu Kunishima, Shunsuke Sato, Manabu Okada, Shuichi Kato, Ryuichi Kato, Hideki Adachi, Masanori Matsukawa, Akio Takayanagi, Naoki Ito, Atsushi Wanifuchi, Takeshi Maehana, Yuki Kyoda, Kohei Hashimoto, Ko Kobayashi, Toshiaki Tanaka, Naoya Masumori

**Affiliations:** ^1^ Department of Urology Sapporo Medical University School of Medicine Sapporo Japan; ^2^ Department of Urology Japan Community Health Care Organization Hokkaido Hospital Sapporo Japan; ^3^ Department of Urology Japanese Red Cross Kushiro Hospital Kushiro Japan; ^4^ Department of Urology Hakodate Goryoukaku Hospital Hakodate Japan; ^5^ Department of Urology Japanese Red Cross Asahikawa Hospital Asahikawa Japan; ^6^ Department of Urology Sunagawa City Medical Center Sunagawa Japan; ^7^ Department of Urology Oji General Hospital Tomakomai Japan; ^8^ Department of Urology Hokkaido Social Work Association Obihiro Hospital Obihiro Japan; ^9^ Department of Urology Steel Memorial Muroran Hospital Muroran Japan; ^10^ Department of Urology Muroran City General Hospital Muroran Japan; ^11^ Department of Urology Saiseikai Otaru Hospital Otaru Japan; ^12^ Department of Urology Takikawa Municipal Hospital Takikawa Japan; ^13^ Department of Urology NTT East Medical Center Sapporo Sapporo Japan; ^14^ Department of Urology National Hospital Organization Hokkaido Medical Center Sapporo Japan

**Keywords:** intravesical recurrence, non‐muscle invasive bladder cancer, radical nephroureterectomy, upper urinary tract carcinoma

## Abstract

**Objectives:**

To evaluate the preoperative risk factors for early intravesical recurrence after radical nephroureterectomy (RNU) in patients with upper urinary tract carcinoma (UTUC) according to the history of non‐muscle invasive bladder cancer (NMIBC).

**Methods:**

We retrospectively evaluated patients who underwent RNU for UTUC between 2012 and 2022 at 14 hospitals. Early recurrence was defined as intravesical pathologically confirmed recurrence within 1 year after RNU. Patients who received single‐dose immediate intravesical instillation (IVI) as prevention for intravesical recurrence were excluded. Using preoperative factors, we examined the risk factors for early intravesical recurrences in patients with and without a history of NMIBC. Death from any cause within 1 year after RNU was regarded as a competitive risk.

**Results:**

We included 504 UTUC patients who were treated with RNU. Among these patients, 126 (25.0%) had a history of NMIBC, whereas 378 (75%) did not. According to multivariate analysis, the presence of macrohematuria, positive cytology in self‐voided urine, and performing diagnostic ureteroscopy prior to RNU were risk factors in patients without a history of NMIBC. On the other hand, an NMIBC diagnosis within 1 year prior to RNU and an extravesical approach for bladder cuff management were risk factors in RNU‐treated patients with a history of NMIBC.

**Conclusions:**

Early intravesical recurrence risk factors differ between UTUC patients with and without a history of NMIBC. Different stratification may be needed to predict intravesical recurrence risk in these two types of UTUC patients.

## 1 Introduction

Intravesical recurrences following radical nephroureterectomy (RNU) pose a significant issue among patients diagnosed with upper urinary tract carcinoma (UTUC). Between 22% and 47% of these patients experience intravesical recurrence, necessitating routine cystoscopy for detection, which can be bothersome for patients [1, 2]. Furthermore, managing these cases often requires transurethral resection, second transurethral resection, and induction or maintenance intravesical instillation (IVI) of chemotherapeutic agents or bacillus Calmette‐Guérin (BCG). Consequently, intravesical recurrence impacts the quality of life and prognosis in some UTUC patients. Therefore, appropriate management based on risk stratification is essential for post‐RNU patients. A history of non‐muscle invasive bladder cancer (NMIBC) is a well‐known risk factor for intravesical recurrence in UTUC patients who have undergone RNU [3, 4, 5, 6, 7]. Hence, it is crucial to analyze the risk factors focusing on this population. However, previous reports have never evaluated the intravesical recurrence risk after RNU based on the history of NMIBC.

Immediate IVI of a single anticancer agent is a well‐established treatment option to reduce the risk of intravesical recurrence post‐RNU in UTUC patients [8, 9, 10, 11]. For instance, Ito et al. reported the efficacy of single‐dose IVI in a phase 2 trial. However, these studies only included UTUC patients without a history of NMIBC [9]. Consequently, it remains unclear whether IVI is as effective for UTUC patients with an NMIBC history as for those without, given that the etiology and mechanisms of intravesical recurrences may differ between these groups. Therefore, this study aimed to investigate the risk factors for intravesical recurrence in UTUC patients, stratified by the presence or absence of a history of NMIBC.

## 2 Materials and Methods

### 2.1 Study Population and Design

Clinical and pathological data of patients who underwent RNU and were pathologically diagnosed with urothelial carcinoma (UC) from January 2012 to December 2022 were retrospectively obtained from 14 hospitals. Exclusion criteria included patients with a history of cystectomy for bladder cancer, lack of follow‐up cystoscopy, incomplete data, non‐UC pathology, and those who received a single IVI after RNU. Cystoscopy was performed every 3 months within the first year post‐RNU to evaluate intravesical recurrence. Given that single‐dose IVI post‐RNU has been reported to have a preventive effect on intravesical recurrence within 1 year, our study focused on early intravesical recurrence, defined as recurrence within 1 year from RNU. All intravesical recurrences were confirmed by pathological diagnosis as UC of the bladder.

The follow‐up period was up to 1 year, and intravesical recurrences beyond this period were not considered in the current study. The time to intravesical recurrence was calculated from the date of RNU to the date of the first pathologically confirmed recurrence. For patients without intravesical recurrence, the follow‐up period was determined from the time of RNU to the last cystoscopic evaluation up to 1 year.

Data collected from medical records included patient background such as gender, age at RNU, smoking status, body mass index (BMI), history of diabetes mellitus or hypertension, history of bladder cancer, presence of preoperative macrohematuria, usage of neoadjuvant chemotherapy, and presence of hydronephrosis. Tumor characteristics included preoperatively determined multiplicity, preoperative self‐voided urine cytology, and prior NMIBC details such as pT stage, tumor size, and period from previous transurethral resection of NMIBC to RNU, particularly for patients with an NMIBC history. Surgical procedure data included lymph node dissection, timing of intraoperative ureteral clipping, method of bladder cuff management (intravesical or extravesical), and whether the procedure was open or laparoscopic. Neoadjuvant chemotherapy and lymph node dissection were not uniformly applied and were decided by the attending clinicians. Patients diagnosed with bladder cancer and UTUC simultaneously were mainly treated by transurethral resection of the bladder tumor prior to RNU. Moreover, these patients were assigned to the UTUC group with a history of NMIBC. The study was approved by the Ethical Committee of Sapporo Medical University School of Medicine (institutional review board number 362–1041) and the ethical committees of each participating institution.

### 2.2 Endpoints and Statistical Analyses

The primary endpoint of the present study was to determine the risk factors for early intravesical recurrence using pre‐RNU factors in newly diagnosed UTUC patients and to stratify them into subgroups based on these risk factors. Statistical differences between groups were assessed using the chi‐square test for categorical variables and Student's t‐test for continuous variables. A *p* < 0.05 (two‐sided) was considered statistically significant. The cumulative incidence of intravesical recurrence rates was examined, and intravesical recurrence‐free survival in the subgroups was compared using Gray's test. To identify factors influencing intravesical recurrence, Fine‐Gray analysis was performed, considering death by any cause within 1 year post‐RNU as a competing risk. The risk factors for early intravesical recurrence in the entire cohort, including those with and without an NMIBC history, were also analyzed by multivariate analysis. All data were analyzed using EZR version 1.35 (Saitama Medical Center, Jichi Medical University) [12].

## 3 Results

We obtained clinical data of 745 patients from 14 hospitals, finally including 504 patients in the analysis. The reasons and number for excluded patients were listed in Figure S1. Table 1 details the preoperative characteristics and intraoperative surgical procedures of the patients, stratified by NMIBC history. Patients with a history of NMIBC had a higher rate of early intravesical recurrence compared to those without NMIBC history. Specifically, 98 early intravesical recurrences (25.9%) were observed in patients without NMIBC history, whereas 50 events (39.7%) were confirmed in those with NMIBC history.

**TABLE 1:** Patient characteristics of the cohort according to the history of non‐muscle invasive bladder cancer.

	NMIBC history (−) *N* = 378	NMIBC history (+) *N* = 126	*p*
Age (median, IQR)	74 (68–79)	75.5 (70–80)	0.187
ECOG‐PS			
PS0	293 (77.5%)	103 (81.7%)	0.205
PS1	74 (19.6%)	17 (13.5%)	
PS2	11 (2.9%)	6 (4.8%)	
Smoking history			
Yes	242 (64.0%)	80 (63.5%)	0.915
No	136 (36.0%)	46 (36.5%)	
Gender			
Female	128 (33.9%)	39 (31.0%)	0.586
Male	250 (66.1%)	87 (69.0%)	
Macrohematuria			
Yes	233 (61.6%)	72 (57.1%)	0.43
No	145 (38.4%)	54 (42.9%)	
Positive cytology at voided urine			
Yes	72 (19.0%)	44 (34.9%)	< 0.001
No	306 (81.0%)	82 (65.1%)	
Preoperative ureteroscopy			
Yes	182 (48.1%)	66 (52.4%)	0.413
No	196 (51.9%)	60 (47.6%)	
Multiple tumors			
Yes	32 (8.5%)	18 (14.3%)	0.084
No	346 (91.5%)	108 (85.7%)	
Tumor lesion			
Renal pelvis	176 (46.6%)	78 (61.9%)	0.002
Ureter	194 (51.3%)	43 (34.1%)	
Both	8 (2.1%)	5 (4.0%)	
Lymph node status			
pN−	99 (26.2%)	32 (25.4%)	0.98
pN+	14 (3.7%)	4 (3.2%)	
pNx	265 (70.1%)	90 (71.4%)	
Surgical approach			
Open RNU	58 (15.3%)	16 (12.7%)	0.561
Laparoscopic RNU	320 (84.7%)	110 (87.3%)	
Timing of ureteral clipping during surgery			
During nephrectomy part	230 (60.8%)	66 (52.4%)	0.149
During distal ureter part	14 (3.7%)	3 (2.4%)	
None	134 (35.5%)	57 (45.2%)	
Management of distal ureter			
Intravesical	86 (22.8%)	43 (34.1%)	0.013
Extravesical	292 (77.2%)	83 (65.9%)	

Abbreviations: ECOG‐PS, Eastern Cooperative Oncology Group performance status; IQR, interquartile range; NMIBC, non‐muscle invasive bladder cancer; RNU, radical nephroureterectomy.

Table 2 presents univariate and multivariate analyses for early intravesical recurrence in UTUC patients without an NMIBC history. Risk factors included the presence of macrohematuria before RNU (HR: 1.80, 95% CI: 1.16–2.81, *p* < 0.01), positive self‐voided urine cytology (HR: 2.25, 95% CI: 1.43–3.53, *p* < 0.001), and pre‐RNU diagnostic ureteroscopy (HR: 1.93, 95% CI: 1.28–2.89, *p* < 0.01). Conversely, in UTUC patients with a NMIBC history, risk factors for early intravesical recurrence included an NMIBC diagnosis within 1 year prior to RNU (HR: 2.19, 95% CI: 1.13–4.27, *p* = 0.02) and extravesical management of the bladder cuff (HR: 2.17, 95% CI: 1.08–4.35, *p* = 0.03) (Table 3).

**TABLE 2:** Uni‐ and multivariate analyses for early intravesical recurrence in patients without history of NMIBC.

	Univariate		Multivariate	
	HR (95% CI)	*p*	HR (95% CI)	*p*
Sex (male)	1.57 (0.99–2.48)	0.052		
Smoking history (yes)	1.62 (1.04–2.52)	0.04	1.56 (0.99–2.44)	0.05
High BMI (22 or more)	0.96 (0.63–1.45)	0.84		
Multiple tumor (yes)	1.59 (0.87–2.80)	0.13		
Macrohematuria (yes)	1.64 (1.05–2.54)	0.03	1.80 (1.16–2.81)	< 0.01
Tumor location (ureter vs. renal pelvis)	1.01 (0.73–1.40)	0.97		
Tumor location (ureter vs. renal pelvis and ureter)	1.31 (0.88–1.93)	0.18		
Hydronephrosis (yes)	1.04 (0.70–1.55)	0.84		
NAC usage (yes)	0.56 (0.26–1.18)	0.13		
Cytology at self‐voided urine (positive)	1.84 (1.18–2.85)	< 0.01	2.25 (1.43–3.53)	< 0.001
Surgical procedure (laparoscopic)	1.17 (0.64–2.12)	0.62		
Bladder cuff management (intravesical)	0.91 (0.56–1.48)	0.71		
Clipping the ureter at nephrectomy part (yes)	1.23 (0.68–1.88)	0.34		
Preoperative ureteroscopy (yes)	1.70 (1.14–2.55)	0.01	1.93 (1.28–2.89)	< 0.01

Abbreviations: BMI, body mass index; NAC, neoadjuvant chemotherapy.

**TABLE 3:** Uni‐ and multivariate analyses for early intravesical recurrence in patients with history of NMIBC.

	Univariate		Multivariate	
	HR (95% CI)	*p*	HR (95% CI)	*p*
Sex (male)	1.73 (0.87–3.44)	0.12		
Smoking history (yes)	0.66 (0.38–1.17)	0.15		
High BMI (22 or more)	0.78 (0.44–1.38)	0.39		
Multiple tumor (yes)	0.83 (0.35–1.98)	0.68		
Macrohematuria (yes)	0.94 (0.54–1.64)	0.84		
Tumor location (ureter vs. renal pelvis)	1.44 (0.83–2.52)	0.19		
Tumor location (ureter vs. renal pelvis and ureter)	1.10 (0.53–2.25)	0.80		
Hydronephrosis (yes)	1.13 (0.65–1.97)	0.67		
NAC usage (yes)	0.70 (0.27–1.79)	0.46		
History of BCG instillation	0.62 (0.31–1.27)	0.17		
Cytology at self‐voided urine (positive)	1.51 (0.88–2.619	0.14		
Surgical procedure (laparoscopic)	0.50 (0.23–1.10)	0.09		
Bladder cuff management (extravesical)	2.00 (1.01–4.00)	0.047	2.17 (1.08–4.35)	0.03
Clipping the ureter at nephrectomy part (yes)	1.28 (0.74–2.23)	0.37		
Prior Bca pT stage (T1)	0.93 (0.52–1.65)	0.81		
Time from prior Bca (less than 1 year)	2.05 (1.06–3.95)	0.03	2.19 (1.13–4.27)	0.02
Preoperative ureteroscopy (yes)	1.17 (0.67–2.03)	0.59		

Abbreviations: BMI, body mass index; Bca, bladder cancer; NAC, neoadjuvant chemotherapy.

**FIGURE 1:**
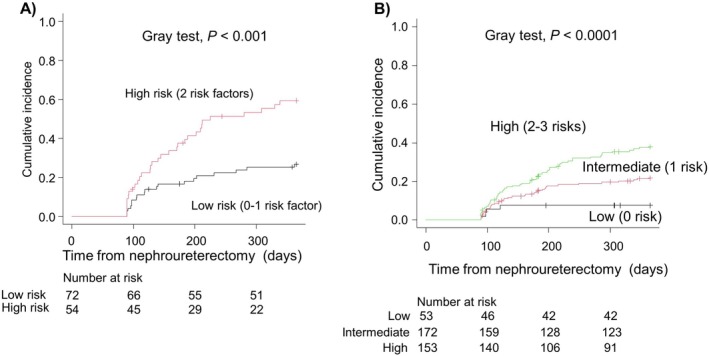
Cumulative incidence of intravesical recurrence after nephroureterectomy according to risk classification in patients (A) with and (B) without a history of NMIBC.

Figure 1 illustrates the cumulative incidence of early intravesical recurrence in both UTUC patient groups based on the number of risk stratifications. Patients without an NMIBC history were stratified into three risk groups: low (no risk factor), intermediate (one risk factor), and high (two or three risk factors). In contrast, patients with an NMIBC history were divided into two risk groups: low (0–1 risk factor) and high (two risk factors). For the entire cohort of 504 UTUC patients, regardless of NMIBC history, risk factors for early intravesical recurrence included male gender (HR: 1.80, 95% CI: 1.28–2.54, *p* < 0.001), positive self‐voided urine cytology (HR: 1.80, 95% CI: 1.28–2.54, *p* < 0.001), performing diagnostic ureteroscopy (HR: 1.56, 95% CI: 1.12–2.15, *p* < 0.01), and a history of NMIBC (HR: 1.48, 95% CI: 1.04–2.09, *p* = 0.03) (Table S1). Among the 504 patients, 22 died within 1 year before experiencing intravesical recurrence, which was regarded as a competing risk in the present study.

## 4 Discussion

In our present study, we aimed to evaluate the individual risk for early intravesical recurrence after RNU. The novel point of this study is the separate assessment of risk factors with and without NMIBC history. Moreover, the design of our study was also significant. First, we only utilized pre‐ or intraoperative RNU factors. Since clinicians need to assess the risk of intravesical recurrence and the need for single‐dose IVI of anticancer agents immediately after RNU, postoperative information such as pathological findings cannot be used. Second, the statistical method considering death by any cause as a competing risk against intravesical recurrence is crucial. Advanced pT stage is considered a risk factor for intravesical recurrence [13]. It is reported that around 25% of pT3 to 4 UTUC patients result in cancer death within 1 year after RNU [14]. Therefore, they have a high risk of death, which may obscure the potential risk of intravesical recurrence. Many studies have evaluated the risk factors for intravesical recurrence without considering this potential competing risk. In our cohort, 22 patients with pT3 to pT4 UTUC died by any cause. Only Somiya et al. evaluated the risk factors for intravesical recurrence using the competing risk concept [15]. It may be reasonable to use this method when evaluating intravesical recurrence, especially when including advanced pT stage UTUC patients in the cohort. Third, because single‐dose IVI reportedly has a preventive effect on early intravesical recurrence [8, 9], we focused on “early” intravesical recurrence. It is important to specify which period of intravesical recurrences are designated as events in clinical studies. We are planning to investigate the preventive effect of single‐dose IVI according to NMIBC history, and therefore, the present study plays a fundamental role in future clinical studies to consider IVI strategy. Conversely, if a study aims to evaluate the risk of intravesical recurrence to consider an appropriate cystoscopic follow‐up protocol, the focus period for recurrence events should be designed longer [16]. Thus, the targeted period of intravesical recurrence and the inclusion of factors, pre‐ or post‐RNU and clinical or pathological findings, should be considered according to what the study aims to evaluate.

There were two randomized clinical trials to evaluate the preventive effect of single‐dose IVI after RNU [8, 9]. The ODMIT‐C trial is a prospective, randomized, non‐blinded trial conducted in 46 British centers between July 2000 and December 2006. In their pre‐protocol analysis, single‐dose IVI of 40 mg mitomycin C demonstrated a preventive effect on 1‐year intravesical recurrence, with rates of 16% for the instillation arm and 27% for the non‐instillation arm. Another trial, the THPMG trial reported by Ito et al. was conducted from 2005 to 2008, including 77 UTUC patients from 11 institutions. In this study, 30 mg of pirarubicin (THP) was used as a single‐dose IVI within 48 h after RNU. At 1‐year post‐RNU, 16.9% of THP‐treated patients and 31.8% of non‐treated patients experienced intravesical recurrence. Therefore, single‐dose IVI is now also recommended in EAU guidelines. However, both trials only included UTUC patients with no history of NMIBC prior to RNU, and it is unclear whether the preventive effect of single‐dose IVI has the same benefit in patients with a history of NMIBC.

A history of NMIBC is a well‐known risk factor for intravesical recurrence after RNU [3, 4, 5, 6]. Therefore, evaluating the risk factors for intravesical recurrence, particularly in these patients, is essential. Furthermore, it is important to explore if IVI demonstrates a similar effect on intravesical recurrence prevention in the future. Lu DD et al. reported a survey on the utilization of IVI in real clinical practice in the United States [10]. It was reported that 15% of clinicians consider IVI if the patients had a history of NMIBC. The rate and reasons for not performing IVI after RNU are also reported. About 50% of clinicians did not consider IVI despite the two randomized trials mentioned previously. The most common reasons were “lack of data” (44%), and 12% “concern for overtreatment (12%).”

According to our findings, the risk factors differ between UTUC patients with and without a history of NMIBC. In patients without an NMIBC history, performing diagnostic ureteroscopy was a risk factor, whereas it was not in patients with an NMIBC history. This fact may indicate that intravesical recurrence is more affected by NMIBC than UTUC characteristics in patients with an NMIBC history. Field cancerization and intraluminal seeding have been proposed as hypotheses for intravesical recurrence after RNU. Given that diagnostic ureteroscopy did not affect intravesical recurrence in patients with a history of NMIBC, field cancerization may be the dominant mechanism for intravesical recurrence in this population, whereas seeding may be the primary mechanism for UTUC patients without a history of NMIBC. If so, the preventive effect of single‐dose IVI on early intravesical recurrence may be limited in these NMIBC‐UTUC patients. Although this clinical issue cannot be solved by our present data, it is an important perspective for conducting future clinical trials to determine who can benefit from IVI according to the history of NMIBC.

The risk factors we demonstrated in our study have been previously reported. For example, macrohematuria prior to RNU was identified as a risk factor for intravesical recurrence. Hashimoto et al. speculated that hemorrhagic tumors can be easily seeded into the bladder [17]. Positive cytology in self‐voided urine was also reported as a risk factor for intravesical recurrence, reflecting a high tumor grade [18]. Although the risk factors themselves are not novel, the differences between the two groups based on the history of NMIBC are novel. Management of distal ureter is also reported as a risk factor for intravesical recurrence after RNU [19, 20]. In meta‐analyses, extravesical approach had a higher intravesical recurrence rate compared to intravesical approach. In our data, extravesical management was a risk factor for intravesical recurrence only in UTUC patients with history of NMIBC. Although the reason is unclear, this may reflect that field cancerization is a more dominant mechanism for intravesical recurrence in UTUC patients with a history of NMIBC rather than intraluminal seeding. If so, a wider margin and complete resection of the ureteral orifice may be needed in this population. Several studies have reported ureteroscopy as a risk factor for intravesical recurrence after RNU. For example, Chen et al. reported that both performing ureteroscopy and biopsy under ureteroscopy increased the risk of intravesical recurrence [21]. Ha et al. reported that flexible ureteroscopy contributed to an increased risk of intravesical recurrence, whereas rigid ureteroscopy did not [22]. In our data, information on the device used (e.g., rigid or flexible ureteroscope) and the data of biopsy were not available. The use of an access sheath for flexible ureteroscopy may be one reason for tumor dissemination to the bladder. It is important to keep in mind that using diagnostic ureteroscopy for suspected UTUC patients may increase the risk of intravesical recurrence. If the tumor in the upper urinary tract is evident and positive cytology is confirmed, omitting ureteroscopy may be an option to avoid future early intravesical recurrence. Takayanagi et al. reported the value of tumor diameter of the upper urinary tract tumor measured by enhanced computed tomography to distinguish malignant tumors from benign ones [23]. According to their report, a tumor diameter of 18 mm was the best cutoff size, with a sensitivity and accuracy of 90.0% and 98.8%, respectively. Although it is important to diagnose malignancy prior to RNU, ureteroscopy should not be routinely performed in some cases. From another perspective, single‐dose IVI after RNU should be strongly considered if the patient underwent diagnostic ureteroscopy. In our study, male gender was an independent risk factor for intravesical recurrence among the entire cohort of 504 patients after RNU (Table S1). Although there was a study indicating vice versa, the male gender was more commonly supported as a risk factor by several reports [7, 24, 25, 26]. Our data could not explain the reason for this gender risk. However, previous studies pointed out the relationship between intravesical recurrence and sex hormones [27, 28]. For instance, Shiota et al. reported that bladder cancer patients who received androgen deprivation therapy had a lower risk of intravesical recurrence. It may be important to consider gender differences in future clinical studies.

Our study has several limitations. Due to its retrospective nature, the management of the distal bladder cuff was not uniformly performed. Moreover, the influence of intravesical recurrence bladder tumors on prognosis was not assessed. It is important to determine if the recurrence affected the patient's prognosis. Two reports from our country demonstrated that intravesical recurrence after RNU might affect the UTUC patient's prognosis if the pT stage of UTUC was not advanced [29, 30]. In our cohort, the number of UTUC patients with a history of NMIBC was relatively small, which may have affected the risk factor evaluation results. However, to the best of our knowledge, this is the first and largest report evaluating intravesical recurrence risk according to a history of NMIBC. In our study, we lacked detailed procedural information for diagnostic ureteroscopy, such as the use of rigid or flexible scopes, ureteral stents, and ureteral stent usage.

In summary, the risk factors for early intravesical recurrence after RNU differ between UTUC patients with and without a history of NMIBC. Therefore, risk stratification, the need for single‐dose IVI, and the preventive effect may differ between these two groups. In terms of prevention, additional clinical trials should be conducted in the future.

## Author Contributions

**Tetsuya Shindo:** conceptualization, methodology, software, data curation, formal analysis, validation, investigation, writing – original draft, writing – review and editing. **Yohei Ueki:** data curation. **Ippei Muranaka:** data curation. **Genki Kobayashi:** data curation. **Shintaro Miyamoto:** data curation. **Yasuharu Kunishima:** data curation. **Shunsuke Sato:** data curation. **Manabu Okada:** data curation. **Shuichi Kato:** data curation. **Ryuichi Kato:** data curation. **Hideki Adachi:** data curation. **Masanori Matsukawa:** data curation. **Akio Takayanagi:** data curation. **Naoki Ito:** data curation. **Atsushi Wanifuchi:** data curation. **Takeshi Maehana:** data curation. **Yuki Kyoda:** conceptualization. **Kohei Hashimoto:** conceptualization. **Ko Kobayashi:** conceptualization. **Toshiaki Tanaka:** conceptualization. **Naoya Masumori:** conceptualization, supervision, writing – original draft, writing – review and editing.

## Consent

This study was conducted using an opt‐out methodology. Participants were informed about the study and were given the opportunity to opt out if they did not wish to participate. Therefore, individual informed consent was not required.

## Conflicts of Interest

Ko Kobayashi and Naoya Masumori are the Editorial Board members of the *International Journal of Urology* and co‐authors of this article. To minimize bias, they were excluded from all editorial decision‐making related to the acceptance of this article for publication.

## Approval of the Research Protocol by an Institutional Reviewer Board

The study was approved by the Ethical Committee of Sapporo Medical University School of Medicine (institutional review board number 362–1041) and the ethical committees of each participating institution.

## Supporting information


**Figure S1.** Details of excluded patients in the present study.

**Table S1.** Uni‐ and multivariate analyses for early intravesical recurrence in entire cohort (*n* = 504).
