# Supplementary material for: Differential Risk Factors for Early Intravesical Recurrence After Radical Nephroureterectomy for Upper Urinary Tract Carcinoma According to the History of Non‐Muscle Invasive Bladder Cancer
Source: Int J Urol. 2025 Feb 13;32(5):567–74. doi: 10.1111/iju.70009 (PMC12022740; doi:10.1111/iju.70009)
Supplement: Supplementary file 1 — Figure S1. Details of excluded patients in the present study. [file IJU-32-567-s001.pptx]

## Slide 1
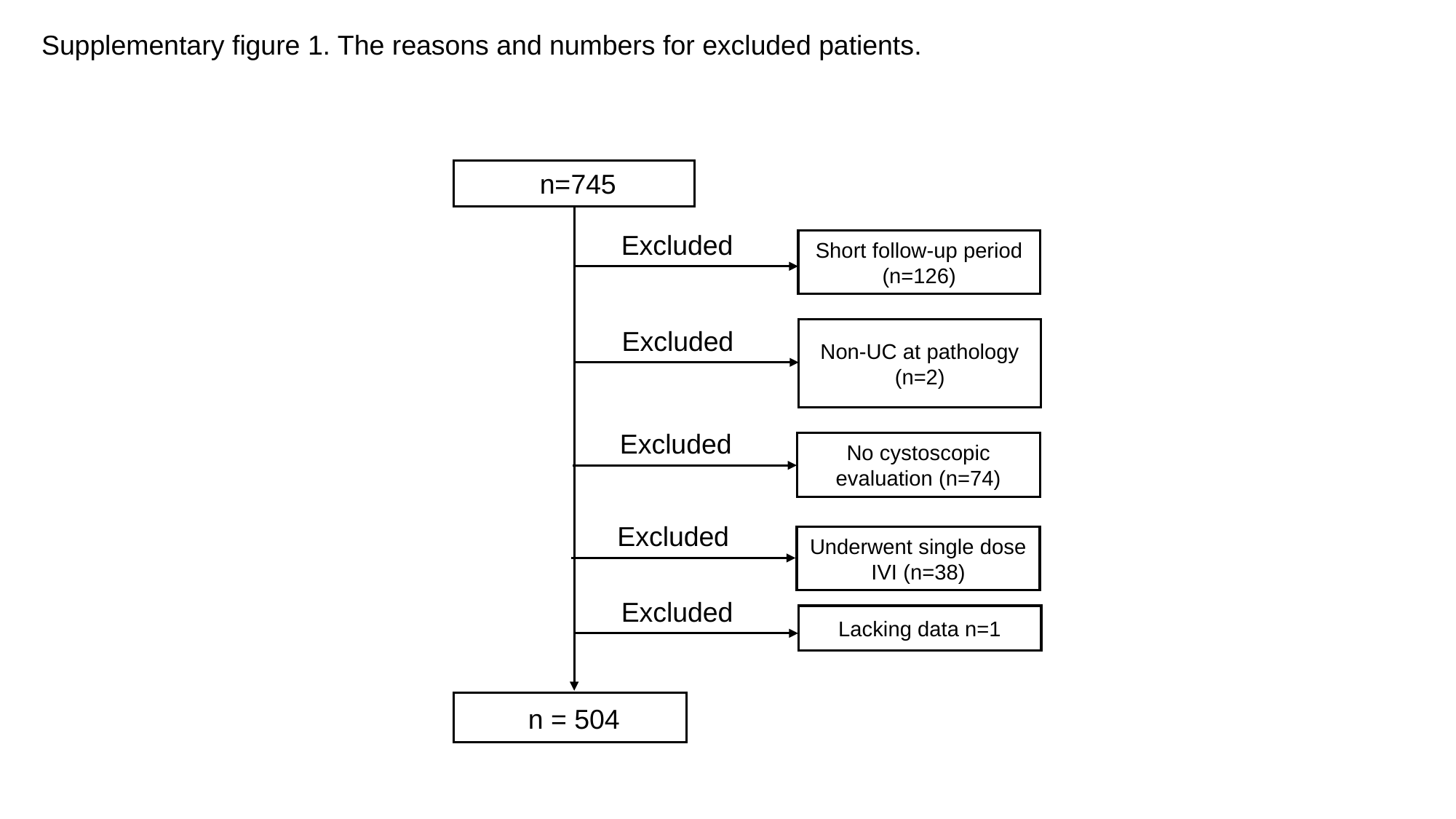

Supplementary figure 1. The reasons and numbers for excluded patients.
 n=745
Excluded
Short follow-up period (n=126)
Excluded
Non-UC at pathology (n=2)
Excluded
No cystoscopic evaluation (n=74)
Excluded
Underwent single dose IVI (n=38)
Excluded
Lacking data n=1
 n = 504
